# Making doctors manage… but how? Recent developments in the Italian NHS

**DOI:** 10.1186/s12913-016-1394-6

**Published:** 2016-05-24

**Authors:** Federico Lega, Marco Sartirana

**Affiliations:** CERGAS - Centre for Research on Healthcare Management and SDA Department of Public Management and Policy, Bocconi University, Milan, Italy; Utrecht University, School of Governance, Utrecht, The Netherlands

**Keywords:** Medical management, Hybrids, Clinical directors, Professionalism, Italy, Hospitals

## Abstract

**Background:**

Involving doctors in management has been intended as one of the strategies to spread organizational principles in healthcare settings. However, professionals often resist taking on relevant managerial responsibility, and the question concerning by which means to engage doctors in management in a manner that best fit the challenges encountered by different health systems remains open to debate.

**Methods:**

This paper analyzes the different forms of medical management experienced over time in the Italian NHS, a relevant “lab” to study the evolution of the involvement of doctors in management, and provides a framework for disentangling different dimensions of medical management.

**Results:**

We show how new means to engage frontline professionals in management spread, without deliberate planning, as a consequence of the innovations in service provision that are introduced to respond to the changes in the healthcare sector.

**Conclusions:**

This trend is promising because such means of performing medical management appear to be more easily compatible with professional logics; therefore, this could facilitate the engagement of a large proportion of professionals rather than the currently limited number of doctors who are “forced” or willing to take formal management roles.

## Introduction

In the health systems of Western countries, clinical management has been intended as one of the strategies to spread New Public Management principles in healthcare settings. Managerial vocabularies became the norm in health policy making, and organizational arrangements that assign relevant leadership responsibilities to doctors, such as the clinical directorate model of hospital organization, were translated in different health systems across the globe [1–3].

This change was not (only) a political fashion, but it was an attempt to respond to current epidemiological and societal changes for which traditional modes of care provision proved inadequate [4–6]. Cost containment needs required the competence and the tools to carefully select investments and to monitor the spending and the use of resources. The progressive rise of chronic diseases and multimorbidities made the hospital organizational model based on disciplinary fragmentation unsuitable for the new challenges and called for an increase in coordination across specialties and professions. Raising patients’ expectations in terms of service quality and timing of care involved service reorganization and a departure from the traditional paternalistic and doctor-centered relationship with patients. Last, the increasing call for accountability required hospitals to provide data on performance, quality, safety, and the efficiency of their operations and to focus increasingly on “value” (effectiveness and appropriateness) in the use of public money. This call determined an evolution from informal and tacit performance management systems, typical of traditional professional organizations, to more explicit and structured procedures to collect, compare and manage data in hospitals, which required a change in doctors’ mode of working with colleagues and administrators [4, 6].

These are some of the factors that contributed to the diffusion of medical management, which provides managerial responsibilities to doctors to make them “hybrids” that are capable of bridging the worlds of medicine and management [7]. However, although there is evidence that clinical management is beneficial for healthcare organizations [8, 9], critics argue that it has not yielded the expected results. As findings across Europe, UK, Sweden, Netherlands, Italy, show, lights and shades characterize the role of doctors as managers: many doctors took managerial roles reluctantly, and few pursued management as an opportunity to develop their professional roles [10–13]. After years of trials, medical management continues to encounter resistance from some medical professionals, and skeptics in different Western countries question whether it is really worth it. Additionally, it was shown that “patterns of accommodation between medicine and management are more nation-specific than is frequently acknowledged” and that existing knowledge of how the processes of doctors’ participation in management “are unfolding across different national systems remains limited” ([14]: 643). Therefore, the question concerning by which means to engage doctors in management in a manner that best fit the contexts and challenges encountered by different countries remains open to debate.

This work’s objective is to contribute to this discussion by highlighting the Italian experience of hybridization and by analyzing alternative solutions experimented to involve doctors in management over time. We will show how new forms of medical management are arising in response to innovations in service provision, following a pathway of development which was largely unplanned, although quite effective. This trend is in accordance with the international attempts to redefine medical professionalism towards a hybrid model capable to encompass managerial values and practices. This paper is structured as follows. We first present research on the different forms of medical management and the contradictions that underlie this concept. Then, we report the illustrative case of the Italian NHS to show the state of the art doctors-in-management roles in hospitals in the last two decades, as well as the recent developments that provide the opportunity for rethinking doctors’ managerial engagement. The results are discussed, and implications for policy and management in Italy and in other health systems are presented.

## Background

In accordance with the healthcare reforms of the last 25 years, a vast body of the scientific and grey literature in the field of healthcare management has investigated doctors in management and their effectiveness [9]. However, medical management has been applied in a heterogeneous set of situations in different healthcare organizational contexts and at different levels within the organization. Therefore, to support effective policy making, it is important to shed some light on the differences which lie within this overarching, but somewhat blurred, concept.

On the one side, we find medical management expressed by physician executives, those hospital CEOs who originate from the ranks of the medical group, as capable of maintaining a dual commitment to the profession and the organization [15, 16]. It was shown how the presence of a doctor as a CEO does have a positive and significant impact on hospital performance [8, 17]. Clinicians can also be involved in the strategic governance of hospitals, and research has shown a positive association between the presence of doctors on hospital boards and hospital effectiveness, in terms of the engagement in quality improvement [18] or perceived quality [19].

Medical management has also been introduced at the middle tier of healthcare organizations, in particular hospitals, particularly with clinical directors, i.e., the medical leads who head large divisions that group different specialties [2, 12]. We found studies that analyze the forms and benefits of this experience in a number of different countries because this model has spread worldwide in recent decades [3, 20–22]. More critical views, which originate from the literature rooted in organization studies and the sociology of professions, have emphasized the problematic sides of this process, notably the resistance from doctors, the subtle co-optation of managerial logics to pursue self or professional interests, the gap between the formal introduction of role and effective managerial role taking, the limits determined by the ambiguity of clinical leader roles as “two way windows”, and the side-effects of contingent managerial authority experienced by doctors-in-management [23–25]. Certain authors have shifted the attention from the individual medical manager to the medical management team, in which competencies are pooled together in a group. This pooling may occur when clinical directorates are led by leadership duos or trios of a clinical director, a nurse manager and an administrative manager. It was shown how these arrangements may reduce doctors’ resistance towards management, making it possible for them to maintain a clinical practice and/or to focus on clinical governance issues [26–28].

A different form of medical management is that performed by frontline professionals, who are increasingly called to engage with improvement initiatives, exerting a leadership that is therefore shared and distributed across the whole organization rather than concentrated at the top management level [29, 30]. This finding is in accordance with the recent studies on medical engagement, i.e., the contribution of all professionals to the enhancement of organizational performance [31]. Achieving a higher engagement of staff was found to have a positive impact on a number of dimensions of organizational outcomes [32]. However, research has shown that the compatibility between formal clinical management structures and a shared leadership approach, in which lower tiers are empowered and managerial responsibilities are distributed, remains puzzling [33]. Furthermore, it was shown that although in principle distributed leadership appeals to professional values and doctors’ desire to be re-empowered, in practice it is not straightforward to effectively engage frontline practitioners with it [34, 35]. The rhetoric of engagement is often strong in the conversation between administrators and doctors-in-management; the practice is much less.

This variety shows how health policymakers and executives continuously attempted to identify specific solutions to bring management to healthcare organizations. Occasionally, solutions were identified through intentional policy interventions, whereas many other times at the organizational level, in search of more effective responses to professional claims, pressures from patients or other stakeholders, and technological innovations. Therefore, medical management is a multifaceted construct, “not one homogeneous process but several, usually very distinct from one another” ([36]: 57), and medical management arrangements are not static but fluid and may evolve over time. This finding calls for an understanding of how schemes of medical management are experimented with to orient research, policymaking and doctors’ training programs. Furthermore, it is important to study experiences outside Anglo-Saxon contexts, where most analysis are still developed. In particular, we analyze the recent developments of medical management of the acute hospital sector in the Italian NHS. The Italian case is theoretically interesting because twenty years ago the country introduced New Public Management reforms aimed at fostering medical management through clinical directorates, as it happened in many other Western health systems. However, over time, and especially in recent years, it has experienced alternative solutions to engage doctors in management, developed at the organizational level rather than in response to system-wide reforms.

Most of the following analysis is based on the research conducted first-hand by the authors and their colleagues for the observatory on the managerialization of the INHS (OASI), which has been run by Bocconi University since 2000. Every year, a specific report is produced, and the topic of management roles and engagement of doctors has been in the agenda of the observatory since its inception. Furthermore, all relevant Italian literature was reviewed.

## Findings from the Italian NHS

Italy has a regionally based National Health Service that provides universal coverage free of charge. Regional governments are responsible for the delivery of primary, secondary and tertiary care services through Local Health Authorities, Public Hospital Trusts and private accredited hospitals. The system is largely public: 78 % of healthcare costs (which, in total, represent 9.1 % of GDP) are publicly funded, and 70 % of hospital beds are public [37, 38]. Regional governments allocate resources to health organizations and have significant degrees of autonomy in organizing the provision of care in terms of health planning, monitoring, determining the number and vocation of health providers, and the cooperation/competition dynamics [39]. Providers, although subject to regional policies, maintain a degree of autonomy in issues such as strategy making, budgetary management or organization. However, many hospital organizational features, including e.g., structures of managerial responsibility, people management rules or the profile of the different professions, are defined at the central level through national laws or national labor collective agreements.

With reference to medical management, specialty unit chiefs have traditionally been the pivotal role in hospital organizations, holding managerial and legal responsibilities over physical resources, medical and nursing staff and strategy making. Since 1992, when the so called “managerialization reform” of the system occurred, the clinical directorate organizational structure was made mandatory for public hospitals in all regions, and practicing physicians, selected among the unit chiefs, were appointed as clinical directors who were (formally) assigned responsibilities over directorate clinical governance, budgeting and strategic decision making [12, 40]. Furthermore, specific managerial education was required for doctors in the position of unit chiefs and above, and general healthcare management training schemes began being promoted by regional governments, as the reform gambled on the further involvement of unit chiefs as doctors in management. The head count and competency level of non-clinical managers was not increased to any significant extent. In the system, a professional group of doctors devoted to hospital hygiene and organization has also been operating for decades. These professionals usually work in a team that supports the hospital Medical Director (who, with the CEO and the Administrative Director, form the top management team). Most Medical Directors originate from the ranks of these hygienists, as well as nearly half of CEOs [41].

Despite the isomorphic adoption of clinical directorate organizational structures, the effective hybridization of doctors was far from being effective in most public Italian hospitals. Only a share of professionals embraced management and effectively changed the manner of working to assimilate managerial logics. The majority resisted such processes, formally accepting clinical director roles but not enacting them in practice according to power vested or expectations attached to the role [28]. Unit chiefs, who had always been clinicians more than managers and who had always advocated the interests of their specialty, proved incapable or unwilling to have strategic and hospital-wide responsibilities. A number of contextual factors contributed to this behavior. For instance, hygienists maintained their traditional pivotal role in addressing hospital organizational matters, and this worked as a “safety net”, which made the engagement of practicing doctors in management less urgent. Additionally, at least in certain cases, hygienists were also directly opposing the spread of medical management, which was viewed as a threat to the previously uncontested turf [42]. Furthermore, the processes of doctors’ hybridization were hampered by the poor support that hospitals provided to clinical directors. In most cases, a part-time nurse manager was paired with the doctor, and eventually a part time secretary. Minimal decentralized support personnel such as operations managers or a finance manager were assigned, and centralized offices (including, for instance, human resources, operations management and logistics, and finance) were composed of personnel with experience in administrative tasks and bureaucratic processes, with minimal competence in management. Therefore, doctors were not exposed to professionals capable of transferring to them a managerial mind-set, and they were required to be involved in small scale problem solving or budgeting analysis, which caused frustration or resistance to management practices. Additionally, there was a lack of delegation from hospital executives; in many hospitals, clinical directors were bypassed and the council of clinical directors never became a real decision making body [12, 28, 40]. Consequently, the in-class managerial training that most doctors undertook was rarely followed by specific opportunities to engage in hospital strategy making [42].

However, in recent years, the Italian NHS has experienced a number of new forms of engagement of doctors in management involving frontline doctors and professionals with middle seniority levels. This change was not the consequence of a policy discourse over healthcare leadership, as the one which took place in the United Kingdom. Instead, it was favored by the introduction of process based organizational structures or innovative hospital layouts, as a number of new hospitals have been re-designed with the aim to pool resources such as operating theatres, wards or outpatient facilities, to favor patient flows and to encourage interdisciplinary collaboration around clinical processes [6, 43]. Because the legally binding structure of responsibilities in hospitals remains strongly centered round unit chiefs and clinical directors, different hospitals autonomously pioneered solutions for the (re)distribution of managerial responsibilities across organizational layers and professions. New responsibilities have been created, such as the direction of clinical pathways which introduce matrix-like organizational forms, or the coordination of clinical network within and across organizations [44, 45]. A number of healthcare organizations are shifting the strategic focus from traditional “vertical” directorates to “horizontal” clinical processes. Pathway coordinators are now provided with goals, responsibilities and budget autonomy, because they often are leading multidisciplinary teams with performance and result-based responsibilities. In other organizations, directors of transversal “centers” are being introduced with the objective of grouping specialties around core product lines, such as care of cancer, women’s health, and trauma [46]. Engaging doctors, including those who are relatively young and without formal hierarchical roles, with these management responsibilities has become a means to train them on the job in management skills, including collaboration, strategy making, goal setting, and performance review. Additionally, engaging doctors has begun to be used as an effective assessment of leadership competencies to be used when formal hierarchical positions are open.

Such major reorganizations have also been accompanied by the conferment of new managerial responsibilities to professionals others than doctors. New roles such as nurse bed managers have been introduced; ward nurse managers have been provided increasing levels of responsibility over technologies and human resources [44]. Responsibilities over operation management and logistics, regarding the management of theatres or patient flows, have been transferred to process engineers or properly trained hygienists, and some have envisioned this as an opportunity to redefine and qualify the vocation of the specialty [47]. These developments have had different outcomes on existing medical management roles, such as unit chiefs and clinical directors. These developments were a major opportunity to reduce the burden of operational tasks and allow top professionals to engage in more qualified managerial activities, particularly strategy creation and clinical governance. However, these changes are being perceived by some as a new attack on medical dominance, the endpoint of a progressive impoverishment of the traditional pivotal role of the unit director [48].

## Discussion

The involvement of doctors in management as clinical directors in the Italian NHS encountered resistance, and did not produce as effective result as expected. A number of doctors proved willing and capable to hybridize, yielding very positive results. However, the majority, years after the introduction of clinical directorates, continue to struggle in the new role. We derived a new understanding of how management competencies are being developed and distributed among different hybrid roles and non-clinical staff. The main trigger for this has been the increase of new forms of medical management that involve frontline professionals and unit chiefs in activities such as decision making over hospital-wide issues, design and management of interdisciplinary and interprofessional care processes, service quality improvement, and the development of clinical networks.

**Fig. 1:**
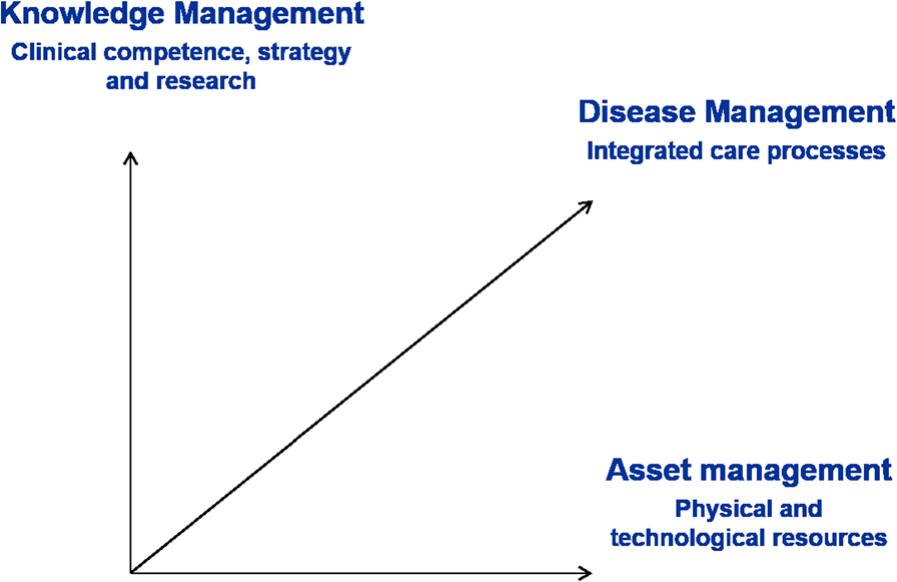
Three axes of responsibility. Adapted from Lega, 2012 [53]

Although traditionally unit chiefs, and to some extent clinical directors, were in full control of staff, physical and financial resources and disciplinary strategy making, a progressive separation of managerial responsibilities is occurring, which can be depicted as in Fig. 1. The *knowledge management* activities, which is the strategy making over the development of medical disciplines, the nurturing of clinical competence, and the planning of research, remains under the control of unit chiefs, senior and respected professionals endowed with status and legitimacy within the medical group. However, the activities related to the establishment, running and improvement of coordinated care processes that provide high quality clinical care to patients, referred to as *disease management*, is increasingly developing and is becoming a responsibility assigned partly to unit chiefs but mainly to frontline doctors, primarily those who are young ad emergent, or certain senior high-caliber consultants. Last, resources such as wards, beds, outpatient services, operating theatres, and technologies, are increasingly shared by a number of different specialties and managed in a coordinated manner. The same applies to non-medical staff, such as nurses or other health staff, which are no longer managed by unit chiefs but are self-governed and work with different medical units. Therefore, the *asset management* is controlled by operation manager roles, an arising group of professionals within the Italian NHS with various backgrounds including engineering, management, nursing and particularly the doctors who specialize in hygiene and hospital organization.

The conceptual framework illustrated by Fig. 1 provides an understanding of new management roles and responsibilities developed in the Italian NHS, and may offer to international scholars new insights regarding how the different dimensions of medical management can be distributed.

Further reflections can be derived from the analysis of the Italian experience, which contributes to the international debate about hybrids and may offer insights to policymakers and practitioners.

Developing clinical management does not necessarily mean to involve professionals in middle or top management roles. New forms of medical management, which we called *disease management*, related to the capacity to understand the functioning of the hospital as a whole, to work and collaborate across disciplines and professional boundaries, to care for the overall performance and quality of the services provided to patients, can be developed. Therefore, management competencies, rather than in the hands of a small group of senior professionals, can be dispersed throughout the organizations. This phenomenon was neither determined by system-level reforms nor accompanied by an explicit narrative on “shared leadership” or “medical engagement”; instead, it emerged as the consequence of the changing work practices and organizational arrangements in healthcare organizations.

We also showed how the professionals engaged in these new roles have a high potential to resolve the contradictions between clinical and managerial logics and understand that clinical leadership does not necessarily regard dismantling professionalism, but rather regards reshaping it. This is in accordance with the work, e.g., of Martin et al. [34] who claim the development of a “new professionalism” characterized by the engagement of clinicians and a focus on coordination, quality improvement and accountability to patient while performing their clinical work. Kirkpatrick and Noordegraaf [49] show how a “reconfigured” professionalism does not compromise traditional values but makes the profession responsive to a wider network of accountability and at the service of the new expectations of patients and society. It is not straightforward for doctors to understand and interiorize this evolution, and this process may be opposed by professionals, as it happened in Italy with unit chiefs, who view this as a new menace to the power of the profession. However, this evolution also has the potential to “rehabilitate professionalism as a force for good, and thus rescue it from the persistent and damaging accusation that it is primarily a self-interested claim aimed at obtaining monopoly rents and other privileges. […] a reinvigorated ‘new professionalism’ […] may embody the best of the professional ethic and secure its place at the heart of service delivery” ([34]: 378–379). Furthermore, this type of development could offer, as it happened in Italy, interesting opportunities to provide visibility and status to a new rank of doctors that has less career development opportunities than in the past due to the economic crisis and reconfiguration processes.

Another interesting remark from the Italian experience concerns the importance of providing organizational support to medical managers. We have seen how a (limited) group of leading professionals can perform effectively in middle or even top management roles. Therefore, such doctors should be identified and provided with adequate training, administrative staff and delegation of responsibilities from executives [28, 42]. Further, these doctors should be paired with nurse managers and other non clinical managers, in order to help them to focus on strategic management and clinical governance issues without jeopardizing their precious time for clinical practice. However, it appears important to develop means to support the development of management competencies for doctors not only when they are close to taking on a formal management role but also since the early stages of their professional career. Despite the popularity of competency models, such as CanMEDS or similar ones, the training of management skills remains underdeveloped [50]. This does not mean to teach a full spectrum of management tools or techniques, such as accounting, financial management or health technology assessment. Instead, the issue is supporting the development of distributed forms of management and leadership, which include taking initiative, understanding how the organization works and feeling a sense of ownership and responsibility for the overall service provided to the patient [51]. The seminal work by Noordegraaf et al. [52] describes a case study that proves the effectiveness of involving medical residents in quality improvement sessions and calls for the development of healthcare research in this area. Interestingly, the study acknowledges that training does not transform all doctors into medical leaders, but that those doctors that “discover they have little affinity with leadership and management […] may acknowledge the importance and difficulties of leading and managing service delivery” ([52]: 21), therefore reducing the resistance to the introduction of managerial logics and principles. This appears to be an interesting path.

Additionally, we found how this evolution in Italy benefited from the support of other professions, particularly nursing but also the discipline of hygienists, who envisioned it as an opportunity for qualification and development. The Italian case shows the benefits that can derive from having a professional class of doctors specialized in hospital hygiene and organization. Although they have contributed to slowing the process of development of clinical directors, there is evidence of the great contributions they can provide to the efficiency and effectiveness of clinical activities [47]. This is something which other health systems could examine.

## Conclusions

This paper has analyzed the developments of hybrid medical management roles focusing on the Italian experience, showing new forms of involvement of doctors in leadership and management activities that are emerging, often without deliberate planning, as a consequence of the innovations in service provision introduced to respond to the changes in the healthcare sector.

We conceptualize this process through a framework that disentangles three axes of management responsibility in healthcare organizations, which may support research and drive the future design of medical managerial roles. We showed that this evolution is promising because these new forms of medical management, especially what we defined them as *disease management*, seems by and large compatible with redefined professional logics. Therefore, medical management appears to be achievable by a large proportion of professionals and not only by a limited number of entrepreneurial doctors willing to take on formal management roles. Furthermore, based on the recent Italian experience, clear directions to improve doctors’ training schemes emerge as well: “When the going gets tough, the tough need to get going.”

Finally, we must acknowledge that this paper has certain limitations. First, after fifteen years of research, there remains a relative scarcity of literature on the topic. Second, in the literature review and in the field experience of the authors there is an overrepresentation of hospitals with higher degrees of managerialization, often located in Northern and Central Italian Regions. Therefore, certain findings may not be equally representative of all Italian regional contexts or healthcare organizations.

### Ethics approval and consent to participate

Not applicable

### Consent for publication

Not applicable

## Abbreviations

CEO: Chief Executive Officer
NHS: National Health Service
